# Quantitative Elastography for Cervical Stiffness Assessment during Pregnancy

**DOI:** 10.1155/2014/826535

**Published:** 2014-03-05

**Authors:** A. Fruscalzo, A. P. Londero, C. Fröhlich, U. Möllmann, R. Schmitz

**Affiliations:** ^1^Obstetrics and Gynecology, Mathias-Spital Rheine, Frankenburgstraße 31, 48431 Rheine, Germany; ^2^Obstetrics and Gynecology, St. Franziskus Hospital, Hohenzollernring 72, 48145 Münster, Germany; ^3^Clinic of Obstetrics and Gynecology, University Hospital of Udine, University of Udine, P.le S. M. Della Misericordia 1, 35100 Udine, Italy; ^4^Clinic of Obstetrics and Gynecology, University of Münster, Albert-Schweitzer Campus 1, 48149 Münster, Germany

## Abstract

*Aim.* Feasibility and reliability of tissue Doppler imaging-(TDI-) based elastography for cervical quantitative stiffness assessment during all three trimesters of pregnancy were evaluated. *Materials and Methods.* Prospective case-control study including seventy-four patients collected between the 12th and 42nd weeks of gestation. The tissue strain (TS) was measured by two independent operators as natural strain. Intra- and interoperator intraclass correlation coefficient (ICC) agreements were evaluated. *Results.* TS measurement was always feasible and exhibited a high performance in terms of reliability (intraoperator ICC-agreement = 0.93; interoperator ICC agreement = 0.89 and 0.93 for a single measurement and for the average of two measurements, resp.). Cervical TS showed also a significant correlation with gestational age, cervical length, and parity. *Conclusions.* TS measurement during pregnancy demonstrated high feasibility and reliability. Furthermore, TS significantly correlated with gestational age, cervical length, and parity.

## 1. Introduction

The process of cervical ripening precedes the active labor by several weeks and is defined as increased softening, effacement, and early cervical dilatation during pregnancy. *Digital cervical examination* remains the standard method for evaluating cervical ripening modifications. The Bishop score is a 10-point scoring system for assessing cervical dilation, effacement, consistency, and position, as well as fetal station [1]. Nonetheless, this approach is subjective and only semiquantitative, and its specificity in patients with low scores is unsatisfactory [2]. Thus, an objective estimation of these parameters, inclusive cervical stiffness assessment, could be very important for the estimation of preterm delivery risk and labor induction success.

*Elastography* is a new imaging technique for the assessment of tissue stiffness by imaging the degree of tissue deformation (i.e., tissue strain, TS) [3, 4]. This tool could allow a quantitative and objective evaluation of the cervical stiffness and thus potentially replace the current subjective and semiquantitative evaluation by palpation [5]. *Tissue Doppler imaging* (TDI) is a Doppler-based tool for the imaging and estimation of tissue strain (TS) using ultrasound. TDI allows for the tracking of tissue movement, while the TDI-Q (Q-quantification) software (Toshiba Medical Systems, Tokyo, Japan) facilitates an estimation of tissue stiffness based on the TS calculation [6]. Two previous preliminary studies have demonstrated the good reproducibility of the method and suggested its possible application for studying tissue elasticity during pregnancy. In these pilot studies, the standardization of the raw data acquisition techniques was addressed and demonstrated [7, 8]. Nonetheless, the feasibility and reliability of TS measurement in all three trimesters of pregnancy have not been investigated yet.

The aim of our study was to evaluate the feasibility and reliability of tissue Doppler imaging- (TDI-) based elastography for cervical quantitative stiffness assessment across all three trimesters of pregnancy.

## 2. Materials and Methods

### 2.1. Patients and Setting

The study was conducted prospectively. It was designed according to the Declaration of Helsinki and was approved by the local ethics board. Informed consent was obtained from all patients at the time of enrolment. The patients included 74 consecutive, unselected women, ranging from the 12th to the 42nd weeks of pregnancy recruited at the University Hospital of Rheine, Germany. The preexamination exclusion criteria included preterm membrane rupture or uterine contractions and a risk of preterm delivery. A minimal functional cervical length of approximately 15 mm (which conformed to the vaginal probe dimensions) was required to provide a sufficient cervical surface for an optimal examination. Indeed, a cervix shorter than the vaginal probe would not permit adequately compressing the cervix.

### 2.2. Strain Measurement

**Figure 1:**
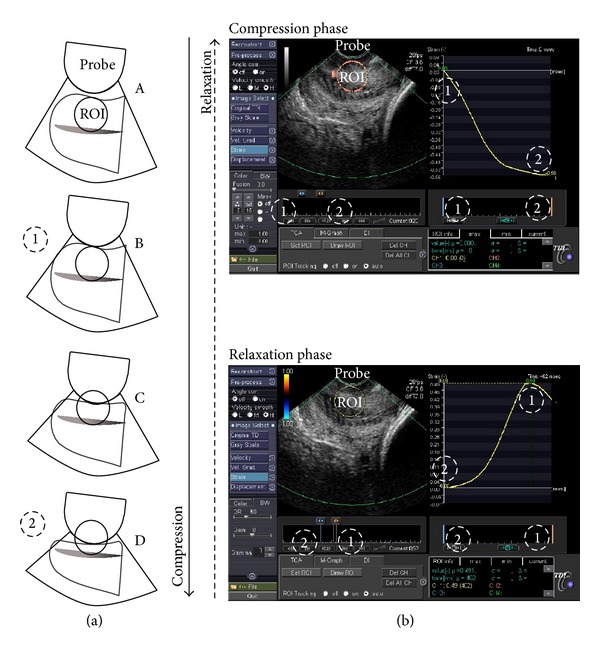
(a) Strain measurement process: the sequence of drawings (A, B, C, and D) shows one cycle of gentle compression and the subsequent relaxation of the anterior lip of the cervix through the vaginal probe, as previously described. (b) The process of TS calculation is displayed (above a compression and under a relaxation phase): a circular region of interest (ROI) is placed covering the whole thickness of the anterior cervical lip. The strain is then calculated during a compression phase (above: from a time of maximal relaxation, 1, to a time of maximal compression, 2), as well as during a relaxation phase (under: from a time of maximal compression, 2, to the time of the subsequent maximal relaxation, 1). The strain values are indicated as a function of the time during the movement (yellow line on the right).

The process of TS measurement was subdivided into the following two steps: the first step consisted of the acquisition of raw data *(raw data set acquisition)* and the second step consisted of the analysis of the acquired raw data, for the purpose of strain measurement (*TS calculation*). The process of strain measurement is described in Figure 1.

### 2.3. Raw Data Set Acquisition

Elastography was performed by a total of 2 gynaecologists. Each patient underwent a transvaginal, real-time elastography by the first operator, and two raw data sets were acquired (f^1^ and f^2^). Elastography was closely replicated by a second operator, and one additional raw data set was acquired (s^1^). Real-time elastography was conducted using a 9 MHz vaginal probe and an Aplio XG ultrasound system (Toshiba Medical Systems, Tokyo, Japan). The procedure was executed as follows [7]: one to two cycles of the gentle compression-relaxation phases were exerted along the longitudinal axis of the cervix, avoiding the lateral and longitudinal dislocation of the tissues, until a maximal compression of its anterior portion was obtained (i.e., until no further shortening of the anteroposterior diameter could be observed and the posterior part of the cervical lip begins to be axially dislocated). In order to check the quality of the movements exerted, the transducer movements could be monitored in the real-time B-mode displayed on the left panel of the screen, while the real-time elastography is contemporarily displayed on the right panel using the split-screen mode. Then, a five-second loop, including the last cycles of compression-relaxation, was acquired and stored in the machine as raw data. The raw data were acquired using TDI software (Toshiba Medical Systems, Tokyo, Japan).

### 2.4. Raw Data Set Analysis and TS Calculation

The TS calculations were performed offline using TDI-Q software. The procedure was executed as follows: the region of interest (ROI) tracking function was selected, and the natural strain preset was chosen with a derivative pitch value of 5 mm. A circular ROI was placed covering the whole thickness of the anterior cervical lip during the frame of the maximal tissue relaxation. Furthermore, the ROI should be placed along the axis of the compressing vaginal probe, in the middle part of the cervix. The TS values were then calculated during the relaxation phase (from the frame of maximal compression to the frame of the subsequent maximal relaxation). Strain values were measured considering the cycle with a larger compression-relaxation cervical tissue excursion. After manually selecting the cycle of interest with a cursor, the software automatically calculated the strain which occurred during the selected movement of compression (Figure 1). The strain calculation was executed by the first operator (F) both on its own acquired raw data set (Ff^1^ and Ff^2^) for the calculation of intraobserver variability and on the first raw data set acquired by the second operator (Fs^1^) for the calculation of interoperator reliability. The TS calculation was then performed by the second operator (S) on its own acquired raw data set (Ss^1^) and on the first raw data set acquired by the first operator (Sf^1^) across the entire patient population, for the calculation of interoperator reliability.

### 2.5. Statistical Analysis

The data were analysed using R (version 2.14.1), and a significance level of *P* < 0.05 was considered to be significant. The data are presented as the median values and the interquartile ranges (IQR), the mean value and standard deviation, with prevalence and absolute values, or the reference values and 95% confidence intervals. First, we performed an analysis for each protocol to test the intraobserver and interobserver reliability of the raw data set acquisition and the TS calculation (per protocol). Then, we conducted additional tests to better assess the reliability of TS measurement (other analysis). For the reliability analysis of the raw data acquisition and TS calculation, we used the following tests: intraclass correlation coefficient (ICC), the mean of differences, and the difference between TS values/mean of TS values (percentage difference). The percentage difference is defined as the difference between two values divided by the average of the two values. It is shown as a percentage and we used it to show the amount of differences between two measurements. Moreover, we used the Bland-Altman plots of the average against the differences of the two measurements, and the limits of agreement were set as two standard deviations from the mean of the differences. We also plotted the 95% confidence intervals of the mean of differences to assess if the no difference line was inside or outside of this range. We also used the following tests for the continuous variables: one way ANOVA, Kruskal-Wallis test, *t*-test, or Wilcoxon test. For the categorical variables, we used Chi-square or exact Fisher tests, where appropriate. To assess the correlations, we used locally weighted scatter-plot smoothing, least squares line, linear regression, and Pearson's test, where appropriate. Finally, we used Levene's test for the homogeneity of variances to assess the degree of variance of differences among the average measurements.

The STARD (Standards for Reporting of Diagnostic Accuracy) criteria for the accurate reporting of studies of diagnostic accuracy were considered [9].

## 3. Results

### 3.1. Population Description

The mean gestational age at the time of examination in the 74 women considered in the confirmatory phase (including the 17 women of trial phase) was 29.08 weeks (±8.93). Of these, 41% had an ongoing pregnancy at the time of the statistical analysis of the study results, whereas 59% had already delivered, all at term of pregnancy and without pregnancy-associated complications. The characteristics of the study population are summarised in Table 1.

### 3.2. Reproducibility of Raw Data Set Acquisition and TS Calculation

**Figure 2:**
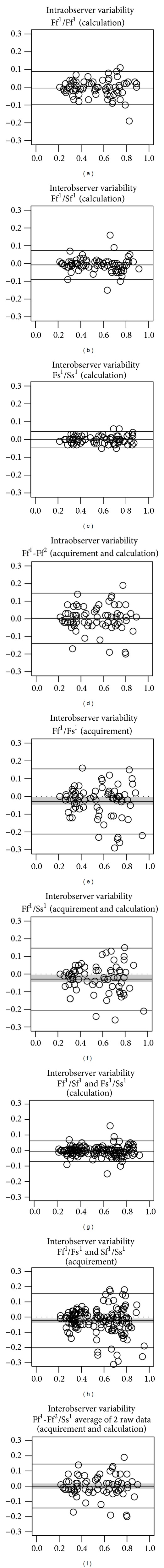
Reliability of tissue strain measurement. Plots (a), (b), (c), (d), (e), and (f) (per protocol analysis): the Bland-Altman plots demonstrate the degree of concordance between the pairs of cervical TSs. The region of agreement is included inside the two standard deviations interval from the mean of differences. The space included between the zero dotted lines and the mean of differences represents the bias. The gray band, where plotted, represents the 95% confidence interval of the mean of differences. F and S refer to the operator undergoing the TS calculation; f and s refer to the operator undergoing the raw data acquirement, where f^1^ and s^1^ and f^2^ and s^2^ refer, respectively, to the first and second raw data sets. (a) The intraobserver variability of the TS calculation of the same raw data (Ff^1^/Ff^1^) is shown. (b) The interobserver variability of the TS calculation of the same raw data by the two investigators (Ff^1^/Sf^1^) is depicted. (c) The interobserver variability of the TS calculation of the same raw data (Fs^1^/Ss^1^) is shown. (d) The intraobserver variability of the raw data acquisition and calculation (Ff^1^-Ff^2^) is shown. (e) The interobserver variability of the raw data acquisition with two raw data sets acquired by different investigators (f and s) and calculated by the same investigator is presented (Ff^1^/Fs^1^). (f) The interobserver variability of the raw data acquisition and calculation by the two investigators is indicated (Ff^1^/Ss^1^). Plots (g), (h), and (i) (other analysis): the Bland-Altman plots demonstrate the degree of concordance between the pairs of cervical TSs. (g) The interobserver variability of the TS calculation: in this plot, we analyse all data from Figures 2(b) and 2(c) (i.e., Ff^1^/Sf^1^ and Fs^1^/Ss^1^). (h) The interobserver variability of the raw data acquirement: the two raw data sets acquired by the two investigators (F and S) are measured by the same investigator (Ff^1^/Fs^1^ and Sf^1^/Ss^1^). (i) The interobserver variability of the raw data acquirement and calculation is shown (Ff^1^-Ff^2^/Ss^1^, yielding an average of the 2 raw data f^1^ and f^2^). In this analysis we took into consideration the average of the raw data measurements of investigator F versus the TS acquisition and measurement of one raw data set by investigator S.

In Table 2, we show the reproducibility of raw data acquisition and the TS calculation during the validation phase. We note that the reproducibility of the TS calculation was high, with an ICC agreement ranging between 0.97 and 0.99. Even if we consider the Bland-Altman plots, most of the differences were within the region of agreement (± two standard deviations) and the average discrepancy between measures (bias: space between dotted zero line and mean of differences line) was inconsistent (Figures 2(a), 2(b), and 2(c) and Table 2). Therefore, we had observed an intra- and interobserver reproducibility of the TS calculation, as well as a high intraobserver reproducibility of the raw data acquisition and the TS calculation (Figure 2(d)). Across all the Bland-Altman plots (Figure 2), there were no significant trends to obtain larger or smaller differences between measurements, as the average increased (*P* > 0.05). Moreover, we tested the variability of the scatter around the bias line across the graph using Levene's test for homogeneity of variance, and the only relevant values are shown in Figures 2(f) and 2(b) (one measurement) and Figure 2(c) (mean of two raw data measurements), which showed significance of *P* = 0.054, *P* < 0.05, and *P* = 0.399, respectively. Furthermore, regarding the interobserver reproducibility of the raw data acquisition (Figures 2(e), 2(f), 2(b), and 2(c) and Table 2), we found the lowest ICC values (ranging between 0.89 and 0.91); however, these values were in the moderate to high agreement regions [10]. If we look at the Bland-Altman plots, we note the presence of a bias (indicated by the white space between the dotted zero line and the mean of the differences line) in all the plots that consider the interobserver variability of raw data acquisition or acquisition and TS calculation (Figures 2(e), 2(f), and 2(b)). Bland-Altman plots the distance between the black line (the mean of differences) and the horizontal middle dotted zero line, and this space is defined as bias (= systematic error) (see Figures 2(e) and 2(f)). The presence of this bias raises the question of whether this average discrepancy between operators (i.e., the bias) is of clinical importance (i.e., if the intra- and interoperator variability of measurements are greater than the variability due to differences among gestational age groups). Unfortunately, we lacked the data to provide such an answer because the clinical usage of this test in the evaluation of cervix has not yet been assessed. Regardless, to evaluate the possible clinical relevance of this average discrepancy between the operators (i.e., the bias), we made some further considerations. First, in Figures 2(e), 2(f), and 2(b), the dotted zero line was out of the 95% confidence interval range of the mean of differences (gray band), while in the other plots, this was not the case. Moreover, in Figure 2(i), considering the mean of the two raw data acquisitions and the TS calculations of F for the comparison, along with the one raw data acquisition and TS calculation of S, the bias was corrected for and Levene's test became nonsignificant. Second, the mean cervical TS at 12–20 gestational weeks was 0.35 (±0.07), at 21–29 gestational weeks was 0.41 (±0.15), at 30–37 gestational weeks was 0.65 (±0.12), and after 37 weeks was 0.73 (±0.09, *P* < 0.05). The difference between every two values of the above TS values in relation to the mean of every two considered values (percentage difference) had a mean value of 43.17% (±24.00%) (CI95 27.05–59.29%), while the percentage difference due to the interobserver variability (D/M of TS, mean, and CI95) had values which ranged between 10 and 12% (Table 2).

### 3.3. Relevant Correlations of Cervical TS with Main Population Features

**Figure 3:**
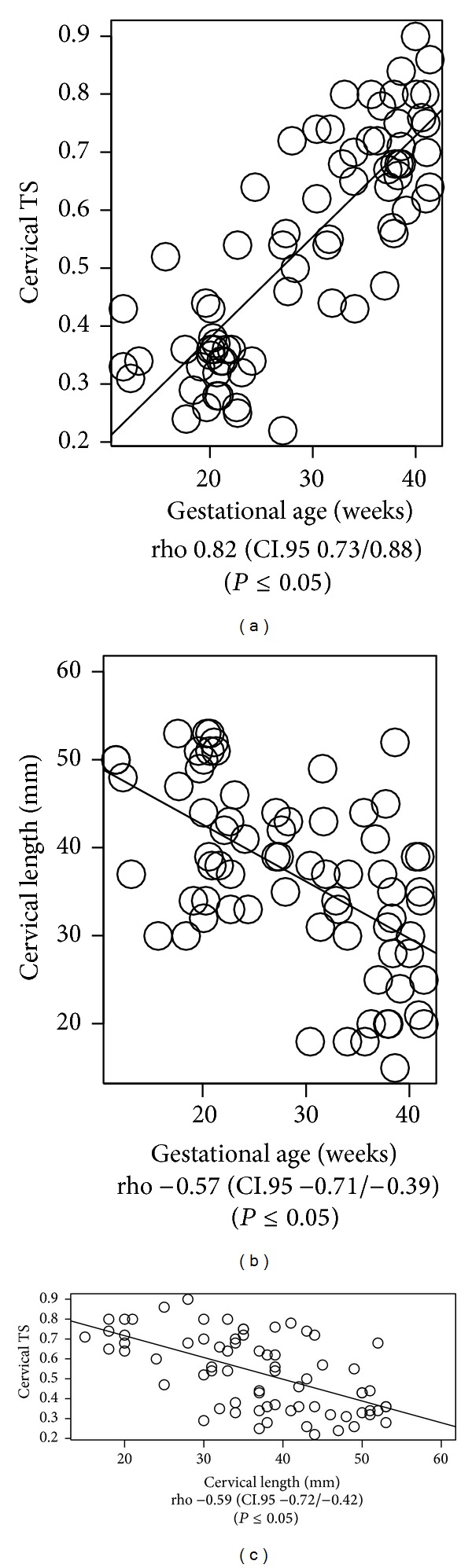
Tissue strain and patients' clinical features. The correlation of TS (average of two raw data set measurements) with the gestational age and cervical length (rho and *P* value refer to Pearson's test).

Finally, we found a significant correlation of the TS with gestational age and cervical length (Figure 3). As full-term pregnancy approached, the TS values increased, reflecting softer cervical tissue. Moreover, a shorter cervical length more frequently correlated with a high TS (again, indicating a softer cervix) (Figure 3). Overall, the pluripara women exhibited shorter and softer cervical properties, compared to the nullipara women (*P* < 0.05). Correlation between TS and cervical length and parity for each subcategory were not significant (except for TS in the 21st to 29th gestational age), probably due to the low number of patients considered in each subgroup (Table 3).

## 4. Discussion

Developing a new imaging technique for an objective assessment of tissue stiffness is potentially an important topic in obstetrics. This could be helpful for an objective and noninvasive description of the physiologic modification of the cervical stiffness occurring during pregnancy and for a better estimation of preterm delivery risk and labor induction success. Other authors tried to address this topic. Tekesin et al. proposed the quantitative ultrasonic tissue characterization (QUTC) tool, a software-based automated tissue gray-scale analysis process. Nonetheless, this tool is based only on a static B-mode picture of the cervix and the insufficient interobserver reliability limited its further development [11]. Recently Swiatkowska-Freund and Preis described a cervical elastography tool where the entity of tissue movements against the vaginal probe during pelvic arterial pulsation and breathings movement were represented on a colour map. Unfortunately, this colour-based Doppler analysis allows only semiquantitative evaluation of tissue movements on a scale from 0 to 4. Furthermore, clinical relevance of these findings has yet to be confirmed [12]. Molina et al. and Fruscalzo and Schmitz proposed a similar approach to that proposed by our group, demonstrating the reliability of quantitative elastography but failing to find the way to standardize the applied pressure needed for translating the tool to the clinical practice [13, 14]. Finally, Hernandez-Andrade et al. demonstrated how, also in a manually generated semiquantitative elastography, cervical tissue strain was related to the most important clinical characteristics of a nonselected population of pregnant women (in particular parity and cervical length). They showed also how cervical tissue strain was more strongly associated with cervical length than with gestational age. This could be explained by the fact that cervical softening, accompanied by cervical shortening, is not always, even if usually, directly related to the advancing gestation age [15]. Similarly, significant correlation between the cervical length and cervical elasticity was reported in their preliminary results by Fuchs et al. [16].

In this study, the natural strain was chosen to test for a universal setting for the TS measurement that could be applied throughout the whole gestational period. Indeed, the Lagrangian, but not the natural strain, failed to perform in the 3rd trimester, when the cervix is softer and the tissue deformation increases [8]. The strain can be calculated both as Lagrangian and natural strain. Whereas the Lagrangian strain describes the deformation (*ε*) of an object with its length *L*(*t*) relative to its initial length *L*(*t*0)(*εL*(*t*) = [*L*(*t*) − *L*(*t*0)]/*L*(*t*0)), the natural strain is based on the temporal integration of the instantaneous deformation (*dε*) of the tissue (*dε* *N*(*t*) = [*L*(*t* + *dt*) − *L*(*t*)]/*L*(*t*)) [17]. In practice, for Lagrangian strain, we just need a start and an end dimension to calculate the strain while natural strain will be calculated over several measurement points. In general, Lagrangian strain is thought to be more accurate than natural strain when small deformations are measured, while the natural strain is thought to be more appropriate in the case of heterogeneous tissues or large tissue deformations (>10–15%). Indeed, the software is less accurate in the strain calculation than Lagrangian strain when following the ROI during large movements. While using natural strain calculation, the measured values are less dependent on the definition of the initial length *L*0 [6]. As discussed in our previous studies [7, 8], in order to standardize the raw data set acquirement process, the compressing force was exerted until a maximal compression of its anterior portion was obtained and the cervical lip begins to be dislocated without further compression. Furthermore, in order to standardize the procedure of strain calculation, the ROI chosen was placed on the full thickness of the anterior cervical lip. Positioning the ROI on different levels of the target tissue (anterior and posterior cervical lip) introduces an important bias on the strain measurement. Indeed, different portions of the cervix are subjected to different forces depending on the distance from the compressing probe (due to the absorption of the force). Otherwise, examining the proximal and distal part of the cervix will imply exerting the compression movements not more perpendicularly to the cervix and introducing a shear strain component that actually cannot be calculated. Furthermore, the distal part of the cervix would slip away during the compression movements, and its lack of stability would compromise the principles of standardisation of the applied force. Finally, a minimal cervical length was required (we proposed a value of 15 mm, fitting the dimension of the vaginal probe) because the compression force should be exerted perpendicular to the longitudinal axis of the cervix. Indeed, it is intuitive that a very short cervix (shorter than the vaginal probe) does not permit adequately compressing the cervix by the probe [7, 8].

The results obtained using the proposed setting (5 mm DP during a relaxation phase) showed excellent results. A high reliability was shown when comparing two measurements of the same raw data set, both if they were calculated by the same operator and if they were calculated by another operator (indicating intra- and interobserver reliability for the TS calculation). These findings indicate that the process of TS calculation using the TDI-Q software has been properly standardised and appears to be stable.

Furthermore, a high reliability was shown for the TS measurements when comparing the three different raw data sets (indicating intra- and interobserver reliability for the whole process of TS measurement). The interobserver reliability was less consistent, due to the biases in raw data acquisition between the two operators. This difference could be overcome, for example, by repeating the measurement twice and calculating the mean TS, so that the eventual bias between operators A and B will disappear. These results confirm the impression that the critical aspect of the TS measurement that accounts for measurement reliability depends upon the process of raw data acquisition.

Finally, our results were correlated with the clinical features of the study population. Specifically, the cervical TS seems to be related both to patient's gestational age and to her cervical length and parity. Therefore, unless the relatively small number of patients used here does not allow for generalisations, these results indicate the potential clinical applicability of the TDI and TDI-Q tools for the quantitative measurement of TS.

### 4.1. Limitations of the Study

A major limitation of the proposed tool is the dependency of TS on the applied force, during the movement of the cervical compression [14, 18]. However, standardised conditions for the raw data acquisition (maximal exertion of compression and care in avoiding the dislocation of the cervix) can optimise the TDI-based TS measurements to enhance feasibility and reliability. The problems of the manual compression and unknown value of the applied force implicate the impossibility to calculate the absolute value of the elastic module of the cervix but allowed an estimation of the tissue stiffness. Recently a manual method for cervical stiffness evaluation was proposed, based on the calculation of the anteroposterior cervical diameter anteroposterior 10 cervical diameter measured before (AP) and after (AP′) application of pressure on the cervix using the transvaginal probe. The author describes an excellent intra- and interobserver correlation, as well as a better prediction of preterm delivery compared to the cervical length measurement [19]. Thus, even if the technique of Parra measures the strain as Lagrangian strain while in our protocol the measure was made using the natural strain, the mechanism used for strain measurement remains the same. Nonetheless, comparison among different studies should be done with caution. Indeed, for a small deformation the natural and Lagrangian strains are similar, as the Lagrangian and natural strains are interrelated by a fixed mathematical formula: (*εN*(*t*) = ln⁡(1 + *εL*(*t*)), but for a larger deformation (as induced in this study), the natural strain will always be greater [17].

The measurement of the stiffness in very short cervices can be limited, due to the impossibility to direct the compressing force perpendicularly to the cervical tissue. However, assessing the risk of preterm delivery in an asymptomatic patient will be probably more interesting than in patients with a very ripened cervix, which are already known to be at great increased risk for preterm delivery.

Furthermore, in order to reduce the number of measurements to be performed, the feasibility and reliability study was first conducted testing some of these combinations among a restricted group of patients by a single operator and then tested among the whole study population. Thus, even if results demonstrated the high reliability of this tool using the chosen preset, this does not exclude that other settings could work even better than this. Further studies considering this topic comparing different settings and ROIs positioned in different regions of the cervix are currently ongoing. The clinical usefulness of this diagnostic tool should be now tested in large prospective clinical settings, even if preliminary results appear to be promising [20].

## 5. Conclusions

Under standardised conditions for the acquisition of raw data and strain calculation, the TDI-based cervical strain measurements obtained during pregnancy are feasible and show high intra- and interoperator reliability. The strain measurements obtained during the relaxation frame using a DP of 5 mm proved to be a well-performing TDI-Q setting for the cervical TS measurement. An objective and quantitative estimation of cervical stiffness during pregnancy could be very important for the estimation of preterm delivery risk and could improve assessment of cervical ripening at term to select patients for successful labor induction. The clinical usefulness of this diagnostic tool should be now tested in large prospective clinical settings.

## Figures and Tables

**Table 1:** Population features.

Gestational age at examination (weeks)	29.08 (±8.93)
First quartile (12–20 weeks' gestation)	25.7% (19/74)
Second quartile (21–29 weeks' gestation)	23.0% (17/74)
Third quartile (30–37 weeks' gestation)	25.7% (19/74)
Fourth quartile (38–42 weeks' gestation)	25.7% (19/74)
Gravidity	2 (1-2)
Parity	1 (1-2)
Para = 0	24.3% (18/74)
Para ≥ 1	75.7% (56/74)
Cervical length (mm)	36.9 (±10.17)
Number of fetuses	
One	97.3% (72/74)
Twins	2.7% (2/74)
Pregnancy outcomes	
Mode of delivery	
Ongoing pregnancy	41% (30/74)
Vaginal spontaneous delivery	38% (28/74)
Cesarean section	20% (15/74)
Operative delivery	1% (1/74)
Gestational age at birth (weeks)	38.8 (±1.8)
Birth weight (grams)	3238.41 (±641.05)

Description of the studied population. The values reported are mean (± standard deviation), median (interquartile range), or prevalence.

**Table 2:** Reliability of tissue strain measurement.

	ICC consistency	ICC agreement	MD	SD	D/M of TS
Reliability of strain calculation					
Intraobserver (Ff^1^/Ff^1^)	0.97 (0.95/0.98)	0.97 (0.95/0.98)	−0.005 (−0.015/0.006)	0.047	7% (5–8%)
Interobserver (Ff^1^/Sf^1^)	0.98 (0.97/0.99)	0.98 (0.97/0.99)	−0.007 (−0.017/0.002)	0.041	5% (4–6%)
Interobserver (Fs^1^/Ss^1^)	0.99 (0.99/1.00)	0.99 (0.99/1.00)	−0.001 (−0.007/0.004)	0.023	3% (3-4%)
Interobserver (Ff^1^/Sf^1^ and Fs^1^/Ss^1^)	0.99 (0.98/0.99)	0.99 (0.98/0.99)	−0.004 (−0.010/0.001)	0.033	4% (3–5%)
Reliability of raw data acquirement					
Interobserver (Ff^1^/Fs^1^)	0.89 (0.84/0.93)	0.89 (0.82/0.93)	−0.029 (−0.050/−0.007)	0.092	12% (9–15%)
Interobserver (Sf^1^/Ss^1^)	0.91 (0.86/0.94)	0.91 (0.85/0.94)	−0.020 (−0.040/0.000)	0.084	10% (8–13%)
Interobserver (Ff^1^/Fs^1^ and Sf^1^/Ss^1^)	0.90 (0.86/0.93)	0.89 (0.85/0.92)	−0.024 (−0.038/−0.009)	0.089	11% (10–13%)
Reliability of raw data acquirement and strain calculation					
Intraobserver (Ff^1^/Ff^2^)	0.93 (0.89/0.96)	0.93 (0.90/0.96)	0.002 (−0.015/0.019)	0.072	10% (8–13%)
Interobserver (Ff^1^/Ss^1^)	0.90 (0.85/0.94)	0.89 (0.82/0.93)	−0.029 (−0.050/−0.008)	0.088	12% (9–14%)
Interobserver (Ff^1^Ff^2^/Ss^1^—average of 2 raw data)	0.93 (0.89/0.96)	0.93 (0.89/0.96)	0.001 (−0.016/0.018)	0.072	10% (8–13%)

Reproducibility of tissue strain measurement, including raw data acquirement and tissue strain (TS) calculation. In brackets the 95% confidence interval is reported. MD: mean of differences; SD: standard deviation of MD; D/M of TS (= percentage difference): difference between TS values/mean of TS values (mean value and CI95).

**Table 3:** Strain values and parity.

	*N *	Para = 0 (*n* = 18)	*N *	Para ≥ 1 (*n* = 56)	*P *
TS	18	0.37 (±0.14)	56	0.59 (±0.17)	<0.05
TS at 12–20 weeks' gestation	8	0.35 (±0.09)	11	0.35 (±0.05)	0.900
TS at 21–29 weeks' gestation	6	0.29 (±0.05)	10	0.49 (±0.14)	<0.05
TS at 30–37 weeks' gestation	3	0.58 (±0.15)	16	0.66 (±0.11)	0.455
TS at 38–42 weeks' gestation	0	NA	19	0.73 (±0.09)	NA
Cervical length (mm)	18	41.47 (±9.92)	56	35.37 (±9.96)	<0.05
Cervical length at 12–20 weeks' gestation	8	43.12 (±10.51)	11	44.55 (±7.26)	0.748
Cervical length at 21–29 weeks' gestation	6	42.67 (±5.35)	10	39.8 (±5.73)	0.334
Cervical length at 30–37 weeks' gestation	3	34.67 (±15.63)	16	32.93 (±9.19)	0.868
Cervical length at 38–42 weeks' gestation	0	NA	19	29.33 (±9.02)	NA

Tissue strain values related to parity and cervical length. Mean (± standard deviation).

## References

[1] Bishop EH (1964). Pelvic scoring for elective induction. *Obstetrics and Gynecology*.

[2] Rozenberg P (2008). The secret cervix. *Ultrasound in Obstetrics and Gynecology*.

[3] Palmeri ML, Nightingale KR (2011). What challenges must be overcome before ultrasound elasticity imaging is ready for the clinic?. *Imaging in Medicine*.

[4] Ophir J, Cespedes I, Ponnekanti H, Yazdi Y, Li X (1991). Elastography: a quantitative method for imaging the elasticity of biological tissues. *Ultrasonic Imaging*.

[5] Eggebø TM, Økland I, Heien C, Gjessing LK, Romundstad P, Salvesen KA (2009). Can ultrasound measurements replace digitally assessed elements of the Bishop score?. *Acta Obstetricia et Gynecologica Scandinavica*.

[6] Sutherland GR, Hatle L, Claus P, D'Hooge J, Bijnens BH (2006). *Doppler Myocardial Imaging: A Textbook*.

[7] Fruscalzo A, Schmitz R, Klockenbusch W, Steinhard J (2012). Reliability of cervix elastography in late first and second trimester of pregnancy. *Ultraschall in der Medizin*.

[8] Fruscalzo A, Steinhard J, Londero AP (2013). Reliability of quantitative elastography of the uterine cervix in at-term pregnancies. *Journal of Perinatal Medicine*.

[9] Bossuyt PM, Reitsma JB, Bruns DE (2003). Towards complete and accurate reporting of studies of diagnostic accuracy: the STARD initiative. *Clinical Chemistry*.

[10] Shrout PE (1998). Measurement reliability and agreement in psychiatry. *Statistical Methods in Medical Research*.

[11] Tekesin I, Hellmeyer L, Heller G, Römer A, Kühnert M, Schmidt S (2003). Evaluation of quantitative ultrasound tissue characterization of the cervix and cervical length in the prediction of premature delivery for patients with spontaneous preterm labor. *The American Journal of Obstetrics and Gynecology*.

[12] Swiatkowska-Freund M, Preis K (2011). Elastography of the uterine cervix: implications for success of induction of labor. *Ultrasound in Obstetrics and Gynecology*.

[13] Molina FS, Gómez LF, Florido J, Padilla MC, Nicolaides KH (2012). Quantification of cervical elastography: a reproducibility study. *Ultrasound in Obstetrics and Gynecology*.

[14] Fruscalzo A, Schmitz R (2012). Quantitative cervical elastography in pregnancy. *Ultrasound in Obstetrics and Gynecology*.

[15] Hernandez-Andrade E, Hassan SS, Ahn H (2013). Evaluation of cervical stiffness during pregnancy using semiquantitative ultrasound elastography. *Ultrasound in Obstetrics and Gynecology*.

[16] Fuchs T, Woyton R, Pomorski M (2013). Sonoelastography of the uterine cervix as a new diagnostic tool of cervical assessment in pregnant women—preliminary report. *Ginekologia Polska*.

[17] D’Hooge J, Heimdal A, Jamal F (2000). Regional strain and strain rate measurements by cardiac ultrasound: principles, implementation and limitations. *European Journal of Echocardiography*.

[18] Fruscalzo A, Schmitz R (2013). Reply. *Ultrasound in Obstetrics and Gynecology*.

[19] Parra-Saavedra M, Gómez L, Barrero A, Parra G, Vergara F, Navarro E (2011). Prediction of preterm birth using the cervical consistency index. *Ultrasound in Obstetrics and Gynecology*.

[20] Fruscalzo A, Londero AP, Frِhlich C, Meyer-Wittkopf M, Schmitz R (2014). Quantitative elastography of cervix for predicting labor induction success. *Ultraschall in der Medizin*.

